# An Exploratory Analysis of the Association Between Catechol-O-Methyltransferase and Response to a Randomized Open-Label Placebo Treatment for Cancer-Related Fatigue

**DOI:** 10.3389/fpsyt.2021.684556

**Published:** 2021-06-29

**Authors:** Teri W. Hoenemeyer, Navneet Kaur Baidwan, Kathryn Hall, Ted J. Kaptchuk, Kevin R. Fontaine, Tapan S. Mehta

**Affiliations:** ^1^Comprehensive Cancer Center, University of Alabama at Birmingham, Birmingham, AL, United States; ^2^Division of Preventive Medicine, Brigham and Women's Hospital, Boston, MA, United States; ^3^Program of Placebo Studies and the Therapeutic Encounter, Beth Israel Deaconess Medical Center and Harvard Medical School, Boston, MA, United States; ^4^Department of Health Behavior, School of Public Health, University of Alabama at Birmingham, Birmingham, AL, United States; ^5^Department of Health Services Administration, School of Health Professions, University of Alabama at Birmingham, Birmingham, AL, United States

**Keywords:** cancer related fatigue, COMT (rs 4680), placebo effect, non-deceptive placebo, COMT rs4818 polymorphism

## Abstract

Previous studies have identified catechol-O-methyltransferase (COMT), as a key enzyme influencing sympathetic function. Although the *COMT* SNP rs4680 and rs4818, are well-studied, little is known about their influence on cancer-related fatigue (CrF) and placebo response. In this study, we examined whether genetic variation in *COMT*, at the functional SNP rs4680 and linked rs4818, influenced open-label placebo (OLP) responses found in cancer survivors reporting moderate to severe CrF. We randomized cancer survivors (*N* = 74) reporting moderate-to-severe CrF to receive OLP or to treatment-as-usual (TAU) and assessed if rs4680 and rs4818 were associated with changes in fatigue severity and fatigue-distressed quality of life. At the end of the initial 21 days, the treatments were crossed over and both groups were re-assessed. Participants with the rs4680 high-activity G-allele (G/G or G/A) or rs4818 C/G genotypes reported significant decreases in fatigue severity and improvements in fatigue-distressed quality of life. The *COMT* rs4818 findings replicated findings in a similar study of OLP in cancer fatigue.

**Clinical Trial Registration:** www.ClinicalTrials.gov, identifier: NCT02522988.

## Introduction

Cancer-related fatigue (CrF), the most common and distressing condition reported by cancer patients, has detrimental effects on physical functioning and quality of life with only marginally effective treatments such as exercise, psychoeducational, erythropoiesis-stimulating agents, Dexamethasone, modafinal, anti-depressants or no treatment (1–11). Over the past 20 years, many biological mechanisms of CrF have been proposed including 5-hydroxy tryptophan (5-HT) dysregulation, cytokine dysregulation, vagal afferent activation, hypothalamic–pituitary–adrenal axis dysfunction, circadian rhythm disruption, and alterations in muscle and adenosine triphosphate (ATP) metabolism (12–15). One causal theory suggests that CrF results from increases in brain serotonin uptake levels that reduce somatomotor drive, modifies hypothalamic-pituitary-adrenal (HPA) axis function and reduces the capacity to perform physical activities (16–18). Several physiological functions are driven by serotonin uptake including the production of 5-HT, a neurotransmitter regulator that regulates somatomotor drive. When serotonin dysregulation happens, it causes disruptions in 5-HT and tryptophan production and synthesis (19). Finally, it is hypothesized that alterations in the autonomic nervous system result in elevated levels of norepinephrine that increase sympathetic activity and alter inflammatory factors (20, 21). As such, genetic variations in catechol-O-methyltransferase (COMT), an enzyme that degrades catecholamines (e.g., norepinephrine, epinephrine, dopamine and catechol estrogens), dysregulates sympathetic function and increases fatigue (22). Although genetic variants of *COMT* single nucleotide polymorphisms (SNP), rs4680 or Val158Met, commonly referred to as the “worrier” (val/A allele) and “warrior” (met/G allele) gene and rs4818, are well-studied and proposed to affect schizophrenia, pain, depression and chronic fatigue, little is known about its effect on CrF, a condition also hypothesized to result from dysregulated sympathetic function (23–28).

In a previously reported randomized controlled clinical trial (RCT) (29) testing the effects of open-label placebos among cancer survivors reporting at least moderate fatigue, we found that, compared to treatment-as-usual, participants who knowingly took 2 placebo pills twice a day for 21 days reported a 29% decrease in fatigue severity and a 39% improvement in fatigue-disrupted quality of life. Here we examine a secondary aim of whether genetic variation in *COMT* at the functional SNP, rs4680 and rs4818 influenced those same OLP responses.

## Materials and Methods

Between August 2015 – May 2017, we conducted a 21-day, single site, two-parallel arm RCT at the University of Alabama at Birmingham's Comprehensive Cancer Center to compare the effects of open-label placebo (OLP) treatment to treatment-as-usual (TAU) among cancer survivors reporting moderate-to-severe CrF. It must be noted that, for this study, TAU was defined as no treatment which is typically the acceptable clinical practice for treating CrF due to limited effective treatments (30). Cancer survivors who completed cancer treatment at least 6 months to 10 years prior to enrollment and who reported at least moderate fatigue (i.e., equal to/>4 on a 0–10 scale) were randomized to OLP (*N* = 39) or TAU (*N* = 35). Potential participants who reported taking prescription medications for CrF, including anti-depression medications, were screened out due to potentially high placebo effects for same consistently reported in the literature (31). Additionally, participants who were originally randomized to OLP treatment group in main study were followed for an additional 21 days after discontinuing treatment and those in the original TAU were offered treatment and followed for 21 days. Participants were compensated $75 for their time.

Seventy-four participants were randomized to take 2 placebo pills twice a day during each 21-day assigned period OLP = 39; TAU = 35). (It must be mentioned that one participant was lost to follow-up after the baseline visit and dropped from subsequent analyses including results reported here). All participants understood that the placebo pill only contained microcrystalline cellulose and not active ingredients. The primary outcomes, measured at baseline, mid-point (21 days) and at completion were self-reported changes in two scales validated for CRF fatigue symptom severity. As a secondary outcome, saliva samples were collected from 72 participants (1 dropped; 1 refused sample) during the baseline visit using the prepIT•L2P purification kit (DNA Genotek) and stored for later analysis. With exception of the results of this sample analysis, details of the methods and results for the primary outcomes have been previously reported (29).

### Site, Ethics Statement and Trials Registration

**Figure 1:**
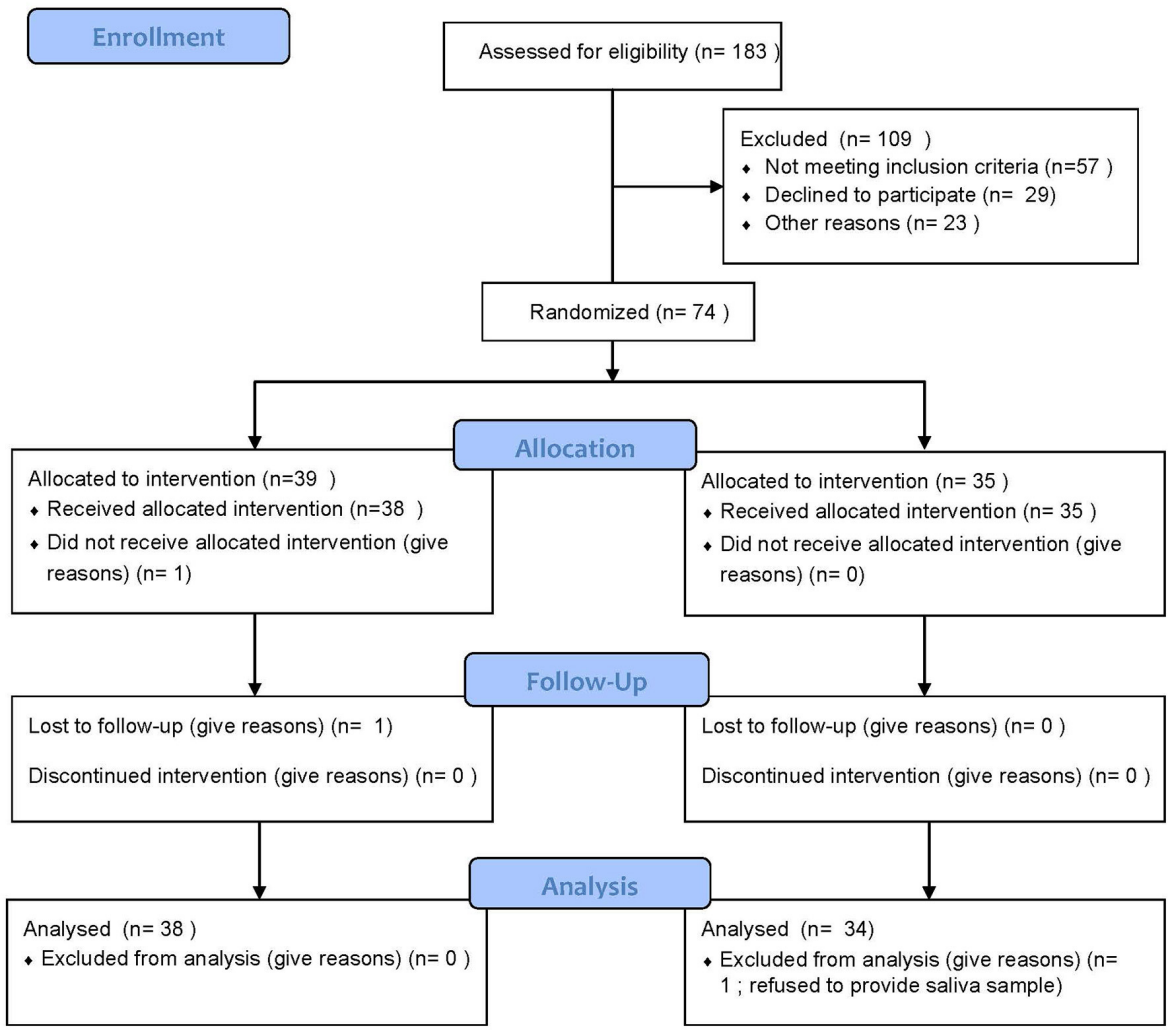
Consort diagram.

This study was conducted in the UAB Comprehensive Cancer Center. The Clinical Trials Review Committee (CTRC) of the Comprehensive Cancer Center and the Institutional Review Board (IRB) of the University of Alabama at Birmingham (UAB) approved this study, and written informed consent was obtained from participants prior to enrollment. The design and procedures of the study were carried out in accordance with the principles of the Declaration of Helsinki. This study was registered with Clinicaltrials.gov on 13/08/15 (NCT02522988). This clinical trial conforms to the CONSORT 2010 Guidelines (Figure 1).

### Study Measures: Primary Outcomes

Demographic information was collected during the initial screening telephone call (e.g., age, race, gender, cancer stage, cancer type, time since last treatment). To assess the effects of OLP on CrF, we used two reliable and well-validated questionnaires, the Fatigue Symptom Inventory (FSI-14) which measures global fatigue symptom severity (FSS) with lower scores indicative of lower fatigue severity. The Multidimensional Fatigue Symptom Inventory Short Form (MFSI-SF30) measures the extent to which fatigue disrupts quality of life (FDQoL) (32, 33). The instrument produces five domain scores: general, physical, emotional, mental health and vigor, as well as a total score. Lower scores are indicative of lower level of fatigue-related disruption of quality of life.

### Secondary Outcome: Genotyping

Saliva samples were collected from 72 (one dropped from study; one refused to provide sample) participants during the baseline visit using the prepIT•L2P purification kit (DNA Genotek), according to the purification protocol, and sent to the Laboratory for Clinical Biochemistry Research located at the University of Vermont for DNA extraction and genotyping. Genotyping of the COMT SNP rs4680 was carried out by PCR amplification followed by automated DNA sequencing on an ABI Prism 3,130 × l Genetic Analyzer (forward primer: GGGGCCTACTGTGGCTACTC, reverse primer: TTTTTCCAGGTCTGACAACG)^24^.

### Randomization and Blinding

Before any participant visits, a research specialist, otherwise uninvolved in the study, placed white sheets of paper with 40 “Group 1” (OLP) and 40 “Group 2” (TAU) assignments into 80 opaque envelopes. The envelopes were shuffled and randomly placed by a graduate student in a pre-enrollment allotment of files assigned consecutive numbers. As each qualified participant agreed to enroll in the study, he or she was assigned a consecutively numbered file containing the concealed group assignment. During the first clinic visit, after the participant was consented and received the placebo orientation (see below), the envelope containing the group assignment was then opened by the participant and assignment arm revealed. Until the envelopes were open, the interaction with participants was identical. All assessments were performed by a research assistant blinded to randomized allocation.

### Power Calculation

Our sample size calculations were based on minimal assumptions. Hence for each outcome, our planned sample size calculations indicated that we would have power of 80% assuming a two-tailed two-sample *t*-test on the outcomes at day 21, Type 1 error rate of 0.05, we would need a sample size of 80 to detect an effect size of 0.64. Techniques such as ANCOVA or two–sample independent *t*-test comparing change scores would be powerful or require less sample size to detect the same effect size depending on the correlation between baseline and follow-up measures.

### Statistical Analysis

This secondary analysis, we used mixed-effects models and analyzed potential associations between the treatment (OLP vs. TAU groups) and genotype (rs4680 and 4818) interactions and post-randomization FSS and FDQoL scores. We also assessed the potential effects of the interaction between treatment and period on the estimate (i.e., mean difference in the scores). We adjusted these analyses for pre-randomization FSS and FDQoL scores and other predictors, including period and demographic variables. In addition, we assessed the potential effects of the interaction between treatment and period on the estimate (i.e., mean difference in the scores), before adjusting for it as a confounder. For all the models, we accounted for the random effects associated with nesting of study IDs and sequence (i.e., sequence in which the treatment was assigned OLP-TAU or TAU-OLP). Statistical significance was tested at the 0.05 level.

## Results

At baseline, 39 participants were randomized to OLP and 35 were randomized to TAU (Table 1). (As previously mentioned, one participant dropped after initial assessment/sample collection and one participant refused to provide a saliva sample. Both were excluded from the analyses). Mean age in the OLP group was about 59, and 56 years in the TAU group. Over two-thirds of the study participants in both groups were White and the majority were females. Of those randomized to OLP who completed the study and provided saliva samples (*N* =37), 45% had the rs4680 genotype A/G (met/val) or G/G (val/val) as did 50% (A/G) and 29% (G/G) of those randomized to TAU group and provided saliva samples (*N* = 34). For the rs4818 genotype category, 29% had the C/C, 47% had the C/G and 24% had the G/G in the OLP group while 56% had C/C, 38% had C/G and 6% had G/G categories in the TAU group.

**Table 1:** Baseline characteristics of the study population by treatment group.

	**Treatment Group**	
**Characteristics**	**OLP mean (SD), *n* (%)**	**TAU mean (SD), *n* (%)**
Age (years)	58.5 (11.4)	56.4 (12.4)
**Race**		
White	29 (76.3)	26 (76.5)
Black	9 (23.7)	8 (23.5)
**Gender**		
Male	11 (29.0)	12 (35.3)
Female	27 (71.1)	22 (64.7)
**rs4680**		
met/met (A/A)	4 (10.5)	7 (20.6)
met/val (A/G)	17 (44.7)	17 (50.0)
val/val (G/G)	17 (44.7)	10 (29.4)
**rS4818**		
C/C	11 (29.0)	19 (55.9)
C/G	18 (47.4)	13 (38.2)
G/G	9 (23.7)	2 (5.9)
**Total**	**38**	**34**

**Figure 2:**
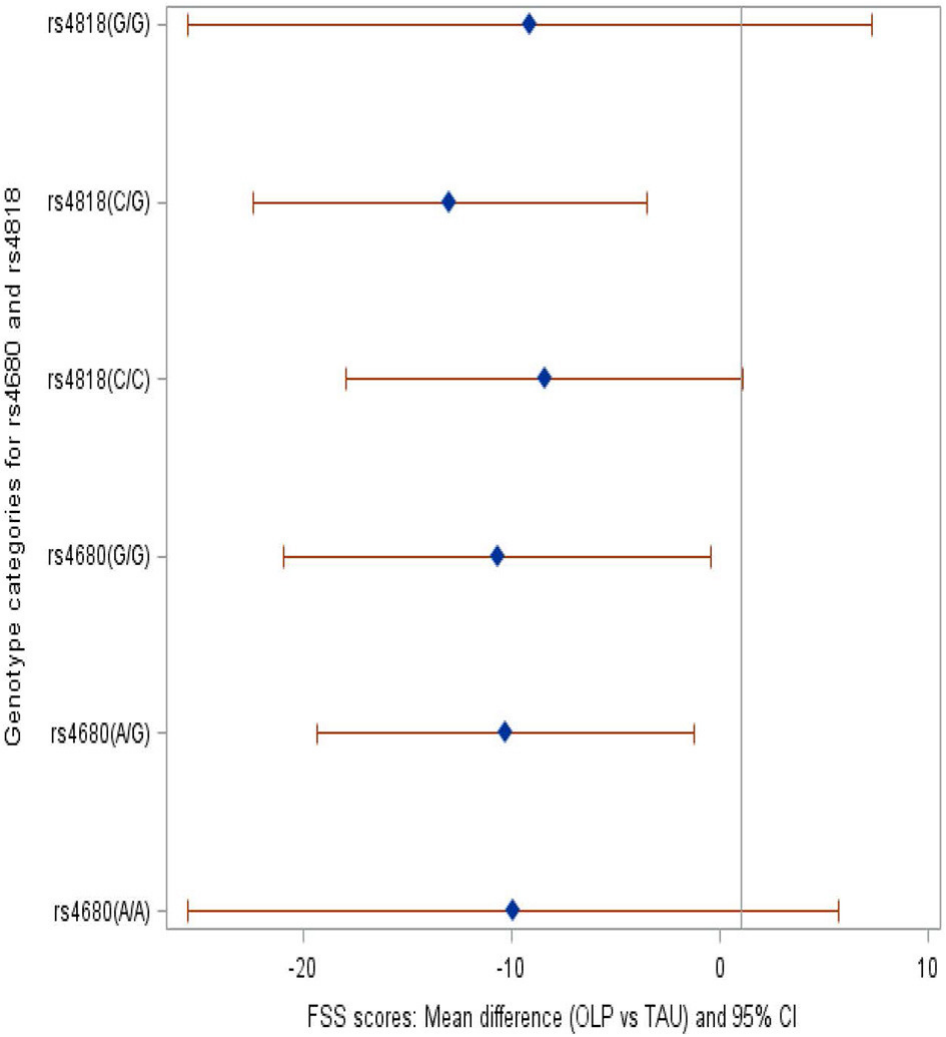
Forest plot showing the mean difference in the FSS scores among the genotype categories within SNPs rs4680 and rs4818 when those randomized to OLP were compared to TAU.

**Figure 3:**
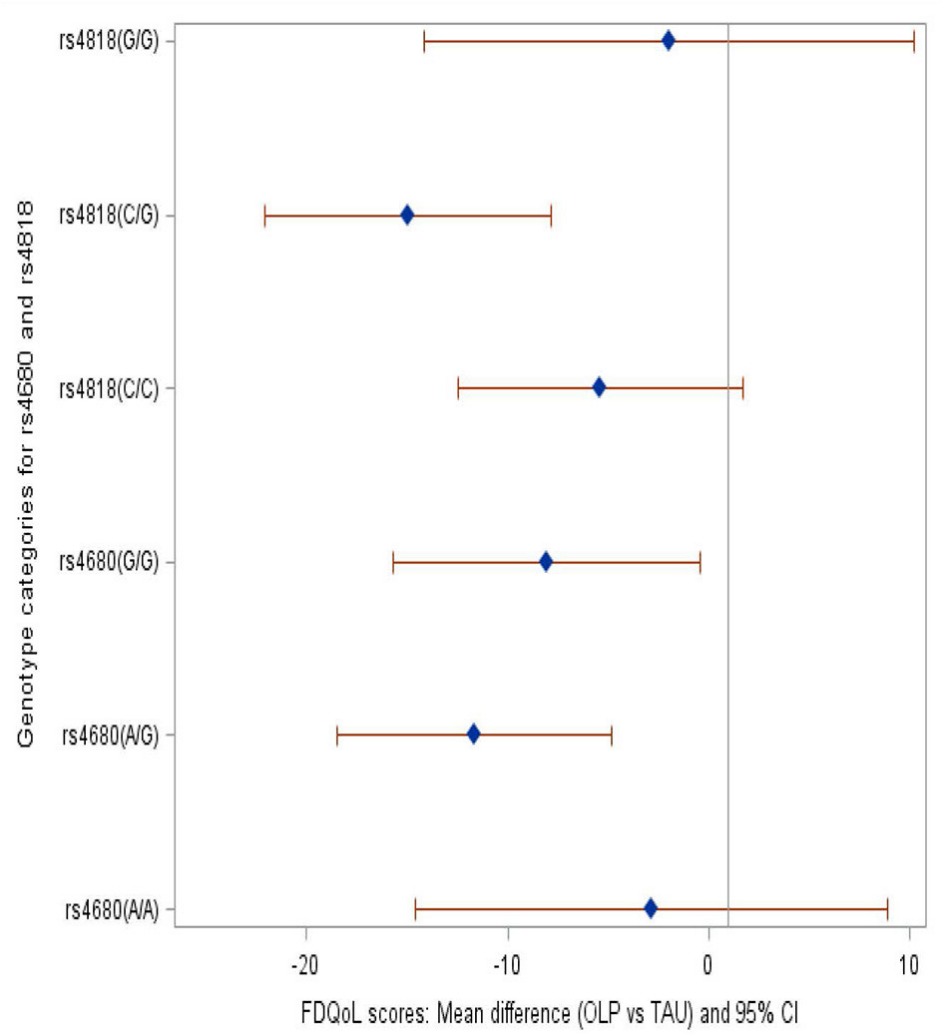
Forest plot showing the mean difference in the FdQoL scores among the genotype categories within SNPs rs4680 and rs4818 when those randomized to OLP were compared to TAU. Brief description of methods [A complete description can be found in (29)].

We compared the OLP group to the TAU group and provide mean differences in FSS and FDQoL scores across the two SNPs: rs4680 and rs4818. Significant decreases were realized in FSS and FDQoL scores among the A/G (met/val) and G/G (val/val) categories for rs4680 and the C/G category for rs4818 for those randomized to OLP compared to TAU. The greatest decreases found in the FSS and FDQoL scores were among the A/G (rs4680) and C/G (rs4818) categories (Table 2, Figures 2, 3). Note that the treatment and period interaction was not significant

Table 2Mean differences in the FSS and FdQoL scores in the OLP group with reference to the TAU group within the rs4680 and rs4818 SNPs.

**Table:** 

	**Effect of treatment on FSS and FdQoL scores among the categories within each SNP**					
	**Fatigue symptom severity (FSS)**			**Fatigue-disrupted quality of life (FdQoL)**		
**Exposure (comparing OLP vs. TAU effect within SNPs)**	**Adjusted: period, pre-treatment scores (Est, 95% CI)**	**+ demographics (Est, 95% CI)**	+treatment*period (Est, 95% CI)	**Adjusted: period, pre-treatment scores (Est, 95% CI)**	**+ demographics (Est, 95% CI)**	+treatment*period (Est, 95% CI)
**rs4680**						
A/A	−9.93	−9.91	−9.91	−2.83	−2.84	−2.84
	(−26.17, 6.30)	(−25.49, 5.66)	(−25. 54, 5.72)	(−14.78, 9.11)	(−14.55, 8.88)	(−14.59, 8.91)
A/G	−10.40^*^	−10.29^*^	−10.30^*^	−11.60^*^	−11.66^*^	−11.67^*^
	(−19.82, −0.97)	(−19.34, −1.25)	(−19.38, −1.23)	(−18.54, −4.66)	(−18.47, −4.85)	(−18.50, −4.84)
G/G	−10.87^*^	−10.64^*^	−10.67^*^	−8.04^*^	−8.05^*^	−8.04^*^
	(−21.52, −0.23)	(−20.86, −0.43)	(−20.92, −0.41)	(−15.83, −0.25)	(−15.68, −0.41)	(−15.69, −0.38)

* *Significant (p < 0.05)*

*Reference: TAU (estimates are for OLP, with reference to TAU within rs4680 genotype)*

*Est: mean difference in scores*

**Table:** 

**rs4818**						
C/C	−8.48	−8.42	−8.42	−5.35	−5.38	−5.38
	(−18.44, 1.47)	(−17.95, 1.12)	(−18.00, 1.15)	(−12.59, 1.89)	(−12.46, 1.71)	(−12.48, 1.73)
C/G	−13.04^*^	−12.97	−12.97^*^	−14.86^*^	−14.95^*^	−14.95^*^
	(−22.90, −3.18)	(−22.41, 3.52)	(−22.46, −3.49)	(−22.10, −7.61)	(−22.03, −7.86)	(−22.06, −7.84)
G/G	−9.57	−9.09	−9.12	−2.11	−1.99	−1.97
	(−26.71, 7.58)	(−25.52, 7.33)	(−25.61, 7.37)	(−14.56, 10.33)	(−14.16, 10.19)	(−14.18, 10.24)

* *Significant (p < 0.05)*.

*Reference: TAU (estimates are for OLP, with reference to TAU within rs4818 genotype)*.

*Est: mean difference in scores*.

We found no statistical significance when the mean FSS and FDQoL scores were compared within each category for rs4680 (A/A, A/G, G/G) and rs4818 (C/C, C/G, G/G) (results not shown). For example, for rs4680, the mean difference in the FSS scores in OLP group with reference to TAU within the A/A category was not significantly different from that in the A/G category.

## Discussion

CrF is a multi-factorial condition that is poorly understood. Relative contributors are hypothesized to include various forms of cancer therapy, and comorbid conditions (e.g., anemia, cachexia, sleep disorders, depression) as well as dysregulation of several physiological and biochemical systems. One mechanism proposed as underlying CrF is the dysregulation of sympathetic and parasympathetic nervous system activity. Specifically, genetic variations in *COMT* (rs4680 and rs4818) have been proposed as degradants of catecholamines dysregulating sympathetic function which increases fatigue (23, 24, 34).

Participants with at least one G-allele in rs4680 (A/G, G/G) and with the rs4818 C/G genotype experienced approximately 10- and 9- point decrease (respectively) in fatigue severity and improvement in fatigue-distressed quality of life. This suggests that specific SNPs, such as rs4680 and rs4818, may influence CrF. However, fatigue-distressed quality of life, only rs4818 C/G participants experienced significant improvements after taking OLP. This finding is consistent with the only other study to look at the association of *COMT* variants with OLP effects on reducing fatigue in cancer survivors (35).

In our previous study, we found that those randomized to OLP reported a 29 and 39% respective improvement in FSS and FDQoL compared to TAU (29). The results of this further analysis seem to align with warrior and worrier hypothesis of the genotype and may have connections with the resilience literature. Previous research efforts suggest that those with val/val genotype have increased COMT activity and thus lower levels of catecholamines, typically labeled as “warriors” (a behavioral phenotype indicating less susceptibility to pain and stress). On the other hand, those with met/met (due to their heightened susceptibility to pain and stress), have been labeled as “worriers” (36, 37). It has been reported that there is an overrepresentation of the CC allele of rs4818 among those with persistent chronic fatigue (20).

An increased occurrence of homozygosity for C allele and wild-type G allele for IL-6-174 (a promoter of reduced plasma levels of IL-6, an inflammatory mediator) has also been reported in fatigued breast cancer survivors while others reported significant cytokine association with CrF among women with breast cancer GG genotypes for TNFα*-*308 (38, 39). As such, polymorphism in neurotransmitter- and HPA axis-related genes associated with COMT variants may have important roles in the etiologies of CrF as it relates to immune dysregulation of cytokines.

Recent research indicates that variation regulation of energy balance and placebo responses may be due to these same genetic variants. Recent studies have demonstrated that genetic variation in the brain's neurotransmitter (e.g., endorphin, cannabinoid, dopamine, and opioid) pathways may modify how a person responds to placebos (40, 41). Genetic variations in the catechol-O-methyltransferase (COMT) gene, which contains an exonic SNP (rs4680) that can reduce its enzymatic activity, can influence the brain's level of the neurotransmitter dopamine and may, thereby, influence the extent of an individual's placebo response (22, 42). Finally, in addition to CrF and chronic fatigue, other studies indicate that G-allele variance may be predictive of more positive treatment effects or less deleterious effects for mood disorders and pain (43–46).

### Implications

The present findings add to this growing body of literature focused on identifying the factors that contribute to symptom susceptibility and burden for cancer survivors. Studies like this exploratory analysis suggest that *COMT* gene variants may be useful in targeting subpopulations with cancer-related fatigue for treatment and symptom management with OLP. In addition, these findings point out different patterns of association of genetic variants for a commonly experienced cancer symptom.

### Limitations

The relatively small sample size restricted our ability to conduct additional analyses comparing each of the categories with the SNPs. Moreover, fatigue is a subjective outcome derived from self-report questionnaires. Next, while case-crossover designs may control for between-person confounders, they may still be within-person confounding that may remain unaccounted for (47, 48).

Another limitation of this study is the relative sensitivity of the HPA system itself. While this study explored hypothesized influences of *COMT* polymorphisms on fatigue, other HPA and autonomic nervous system pathways may have influenced these results. For instance, elevated endogenous levels of 5-HT are known to counteract fatigue and depression, and are highly sensitive to mood changes and stress. Therefore, it could be argued that CrF is not the ideal symptom for testing placebo effects and genetic polymorphisms due to an array of factors (e.g., physical, emotional factors such disease progression, anxiety, stress, depression), that can fluctuate markedly. Additionally, like pain, CrF is accepted as a self-reported condition. As such, objective measures are not currently available and self-reported CrF is susceptible to reporting bias.

## Conclusions

These data provide further evidence about the potential role of genetic loci in placebo response. Both *COMT* rs4680 high-activity G-allele and rs4818 C/G genotype were significantly associated with decreased fatigue severity and improved fatigue-distressed quality of life among cancer survivors participating in a trial of the non-deceptive administration of placebo pills. Although additional work is needed to replicate and elaborate on our findings, these data may provide supportive evidence for a role of genetic loci in open-placebo response and the magnitude of differences between the clinically meaningful improvements found in fatigue severity and fatigue-disrupted quality of life across genotypes.

## Data Availability Statement

The trial protocol and datasets used and/or analyzed during the current study are available from the corresponding author on reasonable request and with the authorization of the University of Alabama at Birmingham's Institutional Review Board.

## Ethics Statement

The studies involving human participants were reviewed and approved by University of Alabama at Birmingham, Institutional Review Board. The patients/participants provided their written informed consent to participate in this study.

## Author Contributions

KH, TJK, and KRF contributed to expertise on trial design, open label placebo and COMT genetic outcomes. TSM contributed as senior author with supervision on genetic outcomes analyses. All authors contributed to the article and approved the submitted version.

## Conflict of Interest

The authors declare that the research was conducted in the absence of any commercial or financial relationships that could be construed as a potential conflict of interest.
