# Supplementary material for: An Exploratory Analysis of the Association Between Catechol-O-Methyltransferase and Response to a Randomized Open-Label Placebo Treatment for Cancer-Related Fatigue
Source: Front Psychiatry. 2021 Jun 29;12:684556. doi: 10.3389/fpsyt.2021.684556 (PMC8275998; doi:10.3389/fpsyt.2021.684556)
Supplement: Supplementary file 1 [file Data_Sheet_1.pdf]

SUPPLEMENTAL INFORMATION: METHODS OVERVIEW

Purpose: The primary purpose of this pilot trial was to evaluate the feasibility and acceptability of using an open-label administration of placebo pills to treat CrF. The secondary purpose was to explore and describe the effects of an open-label administration of placebo pills on CrF Fatigue Distressed Quality of Life (FDQoL) and ___ Fatigue Symptom Severity (GFSS).

- *Specific Aim 1* assesses the feasibility and acceptability of using an open-labeled placebo (OLP) for treating CrF in cancer survivors. ***Hypothesis 1***. An OLP is a feasible and acceptable treatment for CrF. As such, we will meet our target recruitment, enrollment, retention and adherence rates.
- *Specific Aim 2* explores and describes the effects of an OLP treatment on FDQoL and GFSS. ***Hypotheses 2***. Over the course of 49 days, there will be statistically significant improvements in FDQoL and GFSS for participants receiving the OLP treatment.
- *Supplementary aim* explores genetic polymorphisms and response differences.

Target Population. Participants were CSs between the ages of 19 and 70 who had completed cancer treatments between 6 months and 5 years prior, reported a fatigue score of ≥ 4 on a 0-10 scale, and met additional study inclusion/exclusion criteria (see Table 1: Inclusion/Exclusion Criteria).

Study Design. The investigators conducted a 49-day, single site, two-parallel arm, randomized controlled trial to evaluate the feasibility, acceptability and effects of the open-label administration of placebo pills on CRF symptom severity and quality of life for cancer survivors. Study participants were randomized to the intervention or Treatment as Usual (TAU) with a 28-day follow up period in which those assigned to the TAU group were offered the OLP treatment.

Measures were obtained at baseline, 21-days, 28-days and 49-days. (For more details, see Hoenemeyer, et al, 2018).
